# Second-generation antipsychotics and metabolism alterations: a systematic review of the role of the gut microbiome

**DOI:** 10.1007/s00213-018-5102-6

**Published:** 2018-11-20

**Authors:** Karolina Skonieczna-Żydecka, Igor Łoniewski, Agata Misera, Ewa Stachowska, Dominika Maciejewska, Wojciech Marlicz, Britta Galling

**Affiliations:** 1 Department of Biochemistry and Human Nutrition, Pomeranian Medical University, Szczecin, Poland; 2 Sanprobi sp. z o.o. sp. k, Szczecin, Poland; 3 Department of Child and Adolescent Psychiatry, Charité Universitätsmedizin, Berlin, Germany; 4 Department of Gastroenterology, Pomeranian Medical University, Szczecin, Poland; 5 The Zucker Hillside Hospital, Psychiatry Research, Northwell Health,, Glen Oaks, NY USA; 6 Hofstra Northwell School of Medicine, Hofstra University, Hempstead, NY USA

**Keywords:** Microbiota, Second-generation antipsychotics, Metabolism, Dysbiosis

## Abstract

**Rationale:**

Multiple drugs are known to induce metabolic malfunctions, among them second-generation antipsychotics (SGAs). The pathogenesis of such adverse effects is of multifactorial origin.

**Objectives:**

We investigated whether SGAs drive dysbiosis, assessed whether gut microbiota alterations affect body weight and metabolic outcomes, and looked for the possible mechanism of metabolic disturbances secondary to SGA treatment in animal and human studies.

**Methods:**

A systematic literature search (PubMed/Medline/Embase/ClinicalTrials.gov/PsychInfo) was conducted from database inception until 03 July 2018 for studies that reported the microbiome and weight alterations in SGA-treated subjects.

**Results:**

Seven articles reporting studies in mice (experiments = 8) and rats (experiments = 3) were included. Olanzapine was used in five and risperidone in six experiments. Only three articles (experiments = 4) in humans fit our criteria of using risperidone and mixed SGAs. The results confirmed microbiome alterations directly (rodent experiments = 5, human experiments = 4) or indirectly (rodent experiments = 4) with predominantly increased *Firmicutes* abundance relative to *Bacteroidetes*, as well as weight gain in rodents (experiments = 8) and humans (experiments = 4). Additionally, olanzapine administration was found to induce both metabolic alterations (adiposity, lipogenesis, plasma free fatty acid, and acetate levels increase) (experiments = 3) and inflammation (experiments = 2) in rodents, whereas risperidone suppressed the resting metabolic rate in rodents (experiments = 5) and elevated fasting blood glucose, triglycerides, LDL, hs-CRP, antioxidant superoxide dismutase, and HOMA-IR in humans (experiment = 1). One rodent study suggested a gender-dependent effect of dysbiosis on body weight.

**Conclusions:**

Antipsychotic treatment-related microbiome alterations potentially result in body weight gain and metabolic disturbances. Inflammation and resting metabolic rate suppression seem to play crucial roles in the development of metabolic disorders.

**Electronic supplementary material:**

The online version of this article (10.1007/s00213-018-5102-6) contains supplementary material, which is available to authorized users.

## Introduction

Second-generation antipsychotics (SGAs) have been used successfully for the treatment of schizophrenia, bipolar disorders, autism spectrum disorders, major depressive disorders, tic disorder, agitation, sleeping problems, and dementia, among others (Vasan and Abdijadid 2018). The number of prescriptions for SGAs has increased worldwide for both youths and adults (Ilies et al. 2017), with the most recent cross-sectional study of 14 countries finding that quetiapine, risperidone (RIS), and olanzapine (OLZ) are the most frequently prescribed atypical SGAs (Hálfdánarson et al. 2017). An alarming increase in prescriptions, particularly in youth between 15 and 19 years of age, forces us to turn our attention to the health consequences of long-term SGA treatment (Kalverdijk et al. 2017). These consequences include various cardiometabolic adverse effects, such as significant weight gain, hypertriglyceridaemia, hypercholesterolaemia, hypertension, and impaired glucose metabolism (De Hert et al. 2011; Galling and Correll 2015; Galling et al. 2016; Vancampfort et al. 2016), which are all related to metabolic syndrome and cardiovascular disease (Sjo et al. 2017). These changes emerge even after short exposure and increase with cumulative dosages, and differ between agents (Bak et al. 2014).

Overall, in people with severe mental illness, life expectancy is shortened by 10–20 years (Chang et al. 2011), predominantly due to an imbalance in the cardiometabolic system. The prevalence of metabolic syndrome was observed in approximately 30% of patients treated with SGAs (Sanchez-Martinez et al. 2017). Therefore, the American Diabetes Association and the American Psychiatric Association released consensus guidelines to monitor weight and other metabolic parameters in patients treated with SGAs. Moreover, the use of olanzapine in children is discouraged by the Food and Drug Administration because of its association with obesity (American Diabetes Association et al. 2004).

The mechanism of metabolic disruptions, including obesity, hypertension, diabetes, and atherosclerosis (Weiss and Hennet 2017), secondary to SGAs is not fully understood. However, several hypotheses have been proposed, referring to (i) illness- and lifestyle-related factors on metabolism (unhealthy diet, low physical activity, smoking) (Alvarez-Jiménez et al. 2008; Lau et al. 2016; Dayabandara et al. 2017), (ii) SGAs increasing energy intake via neurotransmitter binding in the hypothalamus (Lu et al. 2015), (iii) decreased energy expenditure due to the sedative effect of SGAs (Zimmermann et al. 2003), and (iv) genetic risk associated with the primary illness (Zhang et al. 2016). Other potentially related findings from previous research include (v) diminished insulin synthesis due to the affinity of SGAs for serotonin receptors in the pancreas, leading to diabetic-like metabolic changes (Zhang et al. 2013; Ballon et al. 2014); (vi) elevated muscle, adipose tissue, and liver insulin resistance and glucose transporter efficiency via inhibition of glucose uptake (Dwyer and Donohoe 2003; Verhaegen and Van Gaal 2017); and (vii) accelerated adipose tissue lipogenesis and elevated liver fat content in SGA-treated subjects (Chintoh et al. 2009).

A new approach discussed recently is mediation of SGA-induced adverse effects via the gut microbiota. Maier et al. (Maier et al. 2018) reported that almost one quarter of non-antibiotic drugs used in humans, predominantly antipsychotics, possess antimicrobial activity with potential to imbalance the gut ecosystem. Recently, the inhibition of *Escherichia coli* APC105 growth in vitro with escitalopram was shown as well as its modulatory effects toward other intestinal bacteria in animals (Cussotto et al. 2018). This might mean that the administration of psychopharmacologic drugs may mimic the effect of low-dose antibiotics and thereby be at least partly responsible for antimicrobial resistance of gut microbiota. On the other hand, Nehme et al. reported that atypical antipsychotics, including RIS, OLZ, aripiprazole, clozapine, and quetiapine, would not possess antimicrobial activity, while phenothiazines and thioxanthenes would inhibit the growth of tested bacteria at various minimum concentrations (Nehme et al. 2018).

As dysbiosis may contribute to body weight alterations and cardiometabolic outcomes (Angelakis et al. 2013; Omer and Atassi 2017; Heiss and Olofsson 2017), SGA-induced dysbiosis has been hypothesized to cause adverse metabolic effects (Kanji et al. 2018). In spite of reports linking specific changes in microbiota to weight gain and metabolic disturbances, the subject has not been comprehensively and systematically reviewed, and the mechanism underlying the potential influence of the microbiota on metabolic processes have not been discussed in detail, taking into account limitations regarding study quality.

Therefore, we prepared the first systematic review (SR) investigating the following aims: (1) whether SGAs drive dysbiosis, (2) assessing whether alterations of gut microbiota composition and function affect body weight and metabolic outcome, and (3) examining the possible mechanisms of metabolic disturbances secondary to SGA treatment in rodent and human studies.

## Material and methods

### Search strategy and selection criteria

This study was conducted according to the requirements established in the Preferred Reporting Items for Systematic Review and Meta-Analysis (PRISMA) protocols (Shamseer et al. 2015). Two independent authors (AM and KSZ) systematically searched PubMed/Medline/Embase/PsycInfo/Clinicaltrials.gov from database inception until 03 July 2018. The search was conducted using the following terms identified as medical subject headings (MeSH **bold font**), Supplementary Concept Record terms (SCR *italic font*), and free text terms: (**Microbiota** OR **Gastrointestinal Microbiome** OR microbiome OR microbio*) AND (antipsych* OR neurolept* OR SGA* OR **Antipsychotic agents** OR **Anti-Anxiety agents** OR **Anti-depressive Agents** OR **Anti-depressive Agents**, **Second-Generation** OR **Hypnotics and Sedatives** OR **Antimanic Agents** OR *Olanzapine* OR **Risperidone** OR Atypical Antipsychotics) AND (**Body Weight** OR **Body Weight Changes** OR **Body Weights and Measures** OR **Body Mass Index** OR BMI OR **Metabolism** OR metabolic* OR **Lipids** OR tRMR OR **Cholesterol** OR **Triglycerides** OR **Cholesterol, LDL** OR LDL OR **Fatty Acids** OR **Fatty Acids, Volatile** OR **Acetates** OR **Butyrates** OR **Butyric Acid** OR **Propionates** OR hepatic* OR SCFA OR **Toxins, Biological** OR **Bacterial Toxins** OR **Endotoxins** OR **Lipopolysaccharides** OR **Lipid A** OR **O Antigens** OR LPS OR **Glucose** OR **Insulin** OR HOMA-IR OR **Inflammation** OR **Cytokines** OR **Interleukins**). Reviews, meta-analyses, and systematic reviews were omitted from the search strategy. The electronic search was supplemented by a manual review of the reference lists from eligible publications and relevant reviews.

Inclusion criteria for animal/human studies were as follows:

1. Treatment with SGAs.
2. An in vivo study.
3. A study reporting on metabolic as well as body weight changes and alterations of microbiome composition and function (measured by direct and indirect methods). When an animal or human study consisted of only a few experiments, only experiments fulfilling the above criteria were included and described.

### Data extraction and analysis

At least two authors (AM, KSZ, IŁ) independently extracted information from each study, including details on study characteristics (e.g., study design, treatment protocol, duration, number of subjects, outcome parameters, gut microbiota analysis technique), treatment characteristics (e.g., psychopharmacological agent, dosage, duration of treatment), and subject/patient characteristics (e.g., age, sex, comorbidities, metabolic outcomes). When abstracting data from figures, WebPlot digitizer software was used (https://automeris.io/WebPlotDigitizer/).

The significance of the analysed studies was arbitrarily assigned according to the following scheme: strong—germ-free study, faecal transplantation, statistical significance; middle—conflicting data, lack of relevance due to small group size, data difficult to explain; weak—only co-incidence.

### Risk of bias assessment

Two authors (KSŻ and IŁ) independently assessed the risk of bias using the Systematic Review Centre for Laboratory Animal Experimentation (SYRCLE) Risk of Bias tool for animal studies (Hooijmans et al. 2014), except for item 9 (selective outcome reporting), as this was not assessed in any of the surveyed studies. The STROBE assessment (Vandenbroucke et al. 2014) was used for studies in humans, except for item 16 (main results: unadjusted estimates, confounder-adjusted estimates, category boundaries, translating estimates of relative risk into absolute risk for a meaningful period), as it was not applicable. Outcomes were expressed as the percentage of low-risk judgements (i.e., by dividing the low-risk score by the total number of judgements). When the number was below 16 points (50%), we arbitrarily defined the quality as low. When the results represented up to 60% of the maximum number of points, we treated the study as of moderate quality. Results up to and over 75% were considered high or very high quality, respectively. When a discrepancy occurred, a third author (WM) was involved (Supplementary Figs. S1 and S2).

## Results

### Descriptive data

**Fig. 1:**
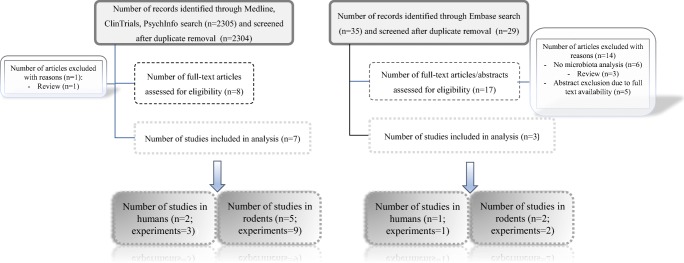
Study flow chart

The initial search yielded 2340 hits; 2315 articles were excluded as duplicates or after evaluation at the title or abstract level. Out of 25 full-text articles that were reviewed, 15 were excluded for not fulfilling the inclusion criteria. Reasons for exclusion were review (*n* = 4), no microbiota analysis (*n* = 6), medications other than SGAs (*n* = 1), and full-text unavailability (*n* = 4), resulting in 10 articles that included 15 experiments in the systematic review (Fig. 1).

### Study and sample characteristics

#### Rodents

Overall, seven articles (experiments = 11) comprising 282 rodents were included: four articles had conducted the experiments using mice (*n* = 198; C57BL/6J) and three using rats (*n* = 84; Sprague–Dawley rats). Rats were of both genders (Davey et al. 2012) or females only (Davey et al. 2013; Kao et al. 2018), aged 6–8 weeks and weighing approximately 200–250 g. Female mice were either 4–8 weeks (Morgan et al. 2014) or 6–7 weeks old (Bahr et al. 2015b), and no information regarding age and gender were found in the other two mouse articles (Grobe et al. 2015; Riedl et al. 2017). Agents tested were RIS (experiments = 6, *n* = 150 rodents) (Grobe et al. 2015; Bahr et al. 2015b; Riedl et al. 2017) and OLZ (experiments = 5, *n* = 132 rodents) (Davey et al. 2012, 2013; Morgan et al. 2014) administered orally (experiments = 7, *n* = 185 rodents) (Morgan et al. 2014; Grobe et al. 2015; Bahr et al. 2015b) or intraperitoneally (experiments = 3, *n* = 84 rodents) (Davey et al. 2012, 2013).

The influence of the microbiota on the metabolic outcome was analysed using the following experimental models: antibiotic usage (experiments = 2) (Davey et al. 2013; Bahr et al. 2015b), high-fat diet (HFD) (Morgan et al. 2014), germ-free model (Morgan et al. 2014), microbiota transfer (Grobe et al. 2015), or SGA treatment prior to cecectomy (Riedl et al. 2017) (one study each). Five rodent protocols were placebo-controlled (Davey et al. 2012, 2013; Grobe et al. 2015; Bahr et al. 2015b; Kao et al. 2018), including one with a faecal transfer trial (Grobe et al. 2015), one study had a cross-sectional design (Morgan et al. 2014), and one described SGA treatment prior to sham operation (Riedl et al. 2017) (for more details, see Table 1).

**Table 1:** Summary of rodent studies

Reference	Subjects: - Age - % Females	Aim, design, and procedure	Number of subjects; duration of intervention	Groups + used substances - Dosage - Administration	Outcome and conclusions
Davey et al. (2012) (Ireland)	Sprague–Dawley rats: - 6 wks - NR	Aim: analysis of the influence of OLZ administration on body weight, behaviour, gut microbiota, and inflammatory and metabolic markers in both gender rats. Design: placebo-controlled (OLZ or VEH). Procedure: Rats were treated with vehicle and two doses of OLZ for 21 days.	*N* = 24; 21 d	OLZ (*n* = 8) - 2 mg/kg twice daily - Intraperitoneal injection B.I.D. OLZ (*n* = 8) - 4 mg/kg twice daily - Intraperitoneal injection B.I.D. VEH (*n* = 8) Distilled water acidified with glacial acetic acid - Twice daily - Intraperitoneal injection	Metabolic: (OLZ vs VEH): 1. ↑ body weight (only in females, higher for lower dose); 2. ↑ food and water intake (mostly in females); 3. ↓ locomotor activity; 4. adipose tissue: ↑ visceral fat, ↓ gene expression of SREBP-1 (in females), ↑ inflammation markers (IL-6 mRNA expression in females and 4-fold increase (insignificant) in males, CD68 expression in females and males); 5. plasma cytokines: ↑ IL-8 and IL-1ß in females, ↓ IL-6 and TNFα in males; 6. ↓ circulating levels of ghrelin in females, ↑ hypothalamic expression of ghrelin 1a receptor mRNA in males. Microbiota: females: 1. ↓ diversity, 2. phyla abundance: *↑ Firmicutes* OLZ 2 mg/kg, 4 mg/kg vs VEH (84.06%, 88.12% vs 72.11%, respectively); ↓ *Actinobacteria* OLZ 2 mg/kg, 4 mg/kg vs VEH (0.34% and 0.15% vs 3.72%, respectively); ↓ *Proteobacteria* OLZ 2 mg/kg, 4 mg/kg vs VEH (0.15% and 0.77% vs 1.60%, respectively); ↓ *Bacteroidetes* OLZ 4 mg/kg 10.88% vs VEH 17.57%. Males: minimal impact of treatment affecting phyla abundance: ↑ *Firmicutes* OLZ 4 mg/kg 91.63% vs VEH 82.66%; ↓ *Proteobacteria* OLZ 2 mg/kg 0.94 vs VEH 3.15%; ↓ *Bacteroidetes* OLZ 4 mg/kg 7.97% vs VEH 14.08%. Conclusion: OLZ treatment is related with weight gain, metabolic disturbances, inflammation and microbiota alteration in gender dependent matter.
Davey et al. (2013) (Ireland)	Sprague–Dawley rats: - 6 wks - 100%	Aim: evaluation if alteration of gut microbiota can play a role in metabolic complications caused by OLZ administration. Design: placebo-controlled. Procedure: After 5 days of lead-in phase with VEH or ABX (to reduce bacteria population in the gastrointestinal tract), rats were randomized to OLZ or VEH treatment lasting 21 days.	*N* = 36/40; 21 d	VEH + OLZ (*n* = 9/10) OLZ: - 2 mg/kg twice daily - Intraperitoneal injection VEH: - Water acidified with glacial acetic acid *-* Intraperitoneal injection ABX + OLZ (*n* = 9/10) OLZ: - 2 mg/kg twice daily - Intraperitoneal injection ABX: - Neomycin (250 mg/kg/day), metronidazole (50 mg/ kg/day), polymyxin B (9 mg/kg/day) - Total vol. 4 mg/kg - Per os VEH + VEH (*n* = 9/10) - Water acidified with glacial acetic acid *-* Intraperitoneal injection ABX + VEH (*n* = 9/10) ABX: - Neomycin (250 mg/day), metronidazole (50 mg/kg/day), polymyxin B (9 mg/kg/day) - Total vol. 4 mg/kg - Per os VEH: - Water acidified with glacial acetic acid - Intraperitoneal injection	Metabolic: (OLZ + VEH): 1. ↑ weight gain; 2. ↑ fat mass; 3. ↑macrophage infiltration of adipose tissue; 4. ↑ free fatty acid release; 5. ↑ hepatic expression of lipogenic enzyme fatty acid synthase (FAS) (effects 1–5 were attenuated by ABX); 6. ↓ insulin sensitivity (direct OLZ effect); 7. ↑ expression of sterol-regulatory element binding protein-1c (SREBP-1c) and acetyl Co-A carboxylase-1 (ACC) (effect of OLZ + ABX treatment). Gut microbiota: 1. OLZ + VEH vs VEH + VEH: A trend toward increased abundance of phylum *Firmicutes* (82.9% vs 76.5%) and reductions of phylum *Bacteroidetes* (10.0% vs 14.3%); 2. OLZ + ABX vs OLZ VEH: a trend toward reduced phylum *Firmicutes* (66.7% vs 82.9%) and increased phylum *Bacteroidetes* (18.9% vs 10.0%). Conclusion: Gut microbiome plays a role in metabolic disturbances caused by OLZ administration
Morgan et al. (2014) (USA)	C57BL/6J mice: - 6 wks - 100%	Experiment no. 1—germ-free study: Aim: testing whether weight gain induced by OLZ treatment in mice having “obesogenic” bacterial profile caused by HFD depends on gut microbiota Design: placebo-controlled study Procedure: group A: germ-free mice—HFD for 7 wks → gut colonization with caecal content from conventionally raised mice → HFD for 9 weeks Group B: germ-free mice HFD + OLZ for 7 wks → colonization → HFD for 2 wks → HFD + OLZ for 7 wks	*N* = 24; 14 wks (7 wks germ-free + 7 wks conventional).	HFD (*n* = 12) - 45% kcal fat - Per os HFD + OLZ (*n* = 12) HFD: - 45% kcal fat - Per os OLZ: - 50 mg/kg of HFD diet - Per os	Metabolic: Germ-free phase: no significant difference in body weight; conventional housing conditions: significant weight gain in the OLZ + HFD group compared to HFD group. Conclusion: gut microbiota was necessary to potentiate weight gain caused by OLZ treatment.
		Experiment no. 2—cross-over study: Aim: studying the influence of OLZ treatment on the weight gain and gut microbiota composition in mice having “obesogenic” bacterial profile induced by HFD. Design: cross-over study Procedure: Group A: 2 wks chow (14 kcal% fat) → 5 wks HFD → 4 wks HFD + OLZ Group B: 2 wks chow → 1 wk. HFD → 4 wks HFD + OLZ → 4 wks HFD	*N* = 24; 4 wks	HFD + OLZ (*n* = 12) HFD: - 45% kcal fat - Per os OLZ: - 50 mg/kg of HFD diet - Per os HFD (*n* = 12) - 45% kcal fat - Per os	Metabolic: 1. Weight gain is more rapid during OLZ ingestion than the placebo phase; 2. adiposity correlated positively with total body weight in OLZ phase; 3. OLZ increased adiposity even after accounting for weight gain. Gut microbiota: 1. decreased alpha diversity, without adjusting for temporal and cohousing effects; 2. increase in the relative abundance of classes *Erysipelotrichi*, *Actinobacteria*, and *Gammaproteobacteria*; 3. decreased abundance of class *Bacteroidia*. *Erysipelotrichi* enrichment due to OLZ treatment was correlated with more rapid weight gain (0.71% increase in weight per 1% increase in abundance). Conclusion: OLZ and HFD have synergistic effect on gut microbiota composition. Relative abundance of some bacteria are associated with more rapid weight gain.
Kao et al. (2018) (UK)	Sprague–Dawley rats - 6–8 wks - 100%	Aim: (1) evaluation of influence of prebiotic [Bimuno™ galactooligosaccharides (B-GOS®)] powder on OLZ-induced weight gain. (2) Testing whether prebiotic can affect mechanism of the action of olanzapine on cortical and hippocampal NMDAR subunit proteins and transcripts. (3) Exploration of the influence of prebiotic and OLZ on the inflammatory as well as metabolic markers and faecal microbiota composition. Design: placebo-controlled. Procedure Group 1: 1 wk water → 2 wks water + saline Group 2: 1 wk B-GOS® → 2 wks B-GOS® + saline Group 3: 1 wk water → 2 wks OLZ + water Group 4: 1 wk B-GOS® → 2 wks B-GOS® + OLZ	*N* = 24; 2 wks	Group 1: Water - Per os Saline - Intraperitoneal injection Group 2: B-GOS®: - 0.5 g/kg/day - Per os Saline - Intraperitoneal injection Group 3: Water - Per os OLZ - 10 mg/kg - Intraperitoneal injection Group 4 (B-GOS®/olanzapine): B-GOS®: - 0.5 g/kg/day - Per os OLZ - 10 mg/kg - Intraperitoneal injection	Metabolic: 1. OLZ—↑ weight gain; 2. B-GOS® prevented weight gain caused by OLZ; 3. ↑ acetate in OLZ and B-GOS® groups and ↓ acetate in Gr. 4; 4. ↑ TNFα in Gr. 3 and 4; 5. ↑ WAT GPR43 mRNA in Gr. 4. 6. No influence of B-GOS® on frontal cortex 5-HT2AR blockade caused by OLZ; 7. ↑ cortical GluN1 protein level in Gr. 4; 8. ↑ cortical GluN2A mRNA in Gr.2; Gut microbiota: Gr. 2 vs Gr. 1: 1. ↑ genus *Bifidobacteria*; 2. ↓ genera: *Escherichia/Shigella* spp., *Coprococcus* spp., *Oscillibacter* spp., *Clostridium Coccoides* spp., *Roseuria Intestinalis* cluster, and *Clostridium* XVIII cluster. No influence of short term OLZ treatment on faecal bacteria composition was observed. Conclusion: Supplementation of B-GOS® to OLZ treatment may prevent weight gain and have favourable effect on cognitive function. Elucidation of mechanism of the influence of B-GOS® on the weight gain caused by short term OLZ treatment seems to be independent on faecal bacteria composition and needs further studies.
Bahr et al. (2015b) (USA)	Wild-type C57BL/6J mice: - 6–7 wks - 100%	Experiment no. 1: Aim: evaluation of the role of the gut microbiota in the development of the weight gain induced by RIS treatment Design: prospective, placebo-controlled. Procedure: Two groups of mice were treated with RIS at two concentrations the third with placebo.	*N* = 15; 60 d	RIS1 (*n* = 5) - 80 μg/d - Per os RIS2 (*n* = 5) - 80 ng/d - Per os VEH (*n* = 5) - Acidified water - Per os	Metabolic: RIS1 vs VEH: weight gain. Gut microbiota (at 58 d): RIS1 vs VEH: ↓ OTUs, ↑ Phyla: *Firmicutes* and *Actinobacteria*, ↑ genera: *Bacteroides*, *Allobaculum*, *Turicibacter*, and *Aneroplasma*, ↓ phyla: *Bacteroidetes* and *Proteobacteria*, ↓ henera: *Alistipes*, *Lactobacillus*, and *Akkermansia*. Conclusion: Higher dose of RIS was associated with weight gain and microbiota alterations.
		Experiment no. 2: Aim: evaluation whether administration of antibiotics, which slightly affect gut microbiota composition, together with RIS, will affect weight gain and energy expenditure. Design: prospective, placebo and verum-controlled. Procedure: Mice were randomized to RIS or VEH-treated group for 48 days. ABX treatment started on the 10th day and continued for 10 days.	*N* = 48; 48 d	VEH (*n* = 8) - Acidified water - Per os Ampicillin (*n* = 8) - 0.54 mg - Per os Ciprofloxacin (*n* = 8) - 0.24 mg - Per os RIS (*n* = 8) - 0.80 μg - Per os RIS + ampicillin (*n* = 8) - 0.80 μg + 0.54 mg - Per os RIS + ciprofloxacin (*n* = 8) - 0.80 μg + 0.24 mg - Per os	Metabolic: Induced weight increase in both RISP and RISP + ABX groups compared to control groups (VEH and both ABX), neither antibiotic significantly changed the influence of RIS on weight gain. No changes concerning food intake, digestive efficiency and energy absorption were observed. Gut microbiota: PcoA of unweight UniFrac distance revealed that ABX had synergistic influence on gut microbiota with RIS. Conclusion: RIS alone is responsible for increased body weight due to decreased energy expenditure.
		Experiment no. 3: Aim: confirmation that weight gain after RIS treatment is associated with gut microbiota changes and decreased energy expenditure using faecal transfer model Design: prospective, placebo-controlled. Procedure: Donor mice: group 1—water ad libitum; group 2—water with risperidone ad libitum (20 mg/ml, *n* = 5) for 9 wks. Faecal material from donor mice was transferred by gavage to naive recipients once daily for 2 wks.	*N* = 22; NA	Donors (9 wks) VEH (*n* = 4): - Water - Per os RIS (*n* = 5): - 20 mg/ml in drinking water - Per os Recipients (14 d) VEH (*n* = 6): - Faecal transfer from VEH donors RIS (*n* = 7): - Faecal transfer from RISP donors (calculated dose of RIS 8.7 ± 1 ng/ml/d)	Metabolic: faecal transplantation from RIS mice—16% reduction in tRMR for recipients due to a reduction in non-aerobic RMR. Conclusion: Microbiota modification after RIS administration is responsible for reduction of non-aerobic RMR.
		Experiment no. 4: Aim: evaluation whether RIS may affect weight gain and energy expenditure due to influence on phageom in the mice gut using phage transfer model Design: prospective, placebo-controlled. Procedure: Phage were isolated from the stool of RIS- and VEH-treated mice and transferred by gavage each day to two groups of mice for 24 days	*N* = 26; NA	Donors VEH (*n* = 6): - NR RIS (*n* = 7): -NR Recipients VEH (*n* = 6): - 7 × 10^9^ phage particles from VEH donors RIS (*n* = 7): - 7 × 10^9^ phage particles from RIS donors	Metabolic: Transfer of phage from RIS-treated donors vs VEH-treated donors caused weight gain, ↓ energy expenditure. Conclusion: Phageome alterations after RIS treatment are sufficient to cause weight gain and decrease energy expenditure.
Grobe et al. (2015) (USA)	C57BL/6J mice: - NR - NR	Aim: confirmation that weight gain after RIS treatment is associated with gut microbiota changes and decreased energy expenditure using faecal transfer model Design: prospective, placebo-controlled. Procedure: donor mice: group 1—vehicle; group 2—RIS. Faecal material from donor mice was transferred by gavage to naive recipients once daily for 2 wks.	*N* = 13; 2 wks	Donors VEH (*n* = NR): - NR RIS (*n* = NR): -NR Recipients VEH (*n* = 6): - Faecal transfer from VEH donors RIS (*n* = 7): - Faecal transfer from RISP donors - 84 ng/d	Metabolic: RIS vs VEH recipients: massive reduction in tRMR. Conclusion: Microbiota modification after RIS administration is responsible for reduction of tRMR.
Riedl et al. (2017) (USA)	C57BL/6J mice: - NR - NR	Aim: analyse whether lack of cecal microbiota can affect RIS influence on tRMR Procedure: In subset of mice used in different experiments, tRMR was measured twice—first time after pretreatment with RIS and second time after cecetomy.	*N* = 26; NR	RIS (*n* = 14) - 80 μg/d - Per os Sham operation (*n* = 12) this group was used also as the control for other experiments	Metabolic: RIS treatment caused significant decrease of tRMR in comparison to the control group. Cecectomy conducted in RIS pretreated mice did not additionally affect tRMR suppression. Conclusion: modulation of microbiota after RIS treatment alone is sufficient to decrease tRMR.

*ABX* antibiotics, *BD* bipolar disorder, *BMI* body mass index, *d* days, *GOS* galactooligosaccharides, *Gr*. group, *HFD* high-fat diet, *HOMA-IR* homeostasis model assessment-estimated insulin resistance, *hs-CRP* high-sensitivity C-reactive protein, *KEGG* Kyoto Encyclopedia of Genes and Genomes, *NR* not reported, *OLZ* olanzapine, *PCoA* principal coordinate analysis, *PICRUSt* Phylogenetic Investigation of Communities by Reconstruction of Unobserved States, *RIS* risperidone, *SCFA* short-chain fatty acid, *SCZ* schizophrenia, *SGA* second-generation antipsychotic, *SOD* superoxide dismutase, *tRMR* total resting metabolic rate, *tx* treatment, *VEH* vehicle, *vs* versus, *wks* weeks, *wk* week, *yrs* years

#### Humans

Overall, three observational studies in humans (experiments = 4; *n* = 232) including two cross-sectional groups and two longitudinal groups were included (Bahr et al. 2015a; Flowers et al. 2017; Yuan et al. 2018), all aiming to assess whether RIS (*n* = 74) (Bahr et al. 2015a; Yuan et al. 2018) or mixed SGAs (*n* = 117) (Flowers et al. 2017) would affect the microbiota composition and, consequently, metabolic indices. One article included 33 male children (mean age: cross-sectional group, 12.2 ± 2.5 years; longitudinal group, 11.7 ± 1.1 years; no treatment, 12.0 ± 1.8 years) (Bahr et al. 2015a). In addition to chronic RIS treatment, patients were also administered psychostimulants (100%), α-2 agonists (66%), and selective serotonin reuptake inhibitors (SSRIs, 11%), whereas controls did not receive antipsychotics but were taking psychostimulants (70%), α-2 agonists (30%), and SSRIs (20%). Another study involved 117 adults (study group treated with SGAs, 34 females and 12 males aged 46 ± 12 years; control group, 48 females and 21 males aged 51.7 ± 13.5 years) (Flowers et al. 2017). Co-administration of antidepressants (53%), mood stabilizers (57%), lithium (22%), and benzodiazepines (39%) was identified in SGAs and the control group. The last study evaluated RIS-induced metabolic parameters such as antioxidant superoxide dismutase (SOD) and high-sensitivity C-reactive protein (hs-CRP) in relations to microbiota composition between drug naïve 41 schizophrenia (SCZ) patients (18 females, 23 males; mean age 23.1 ± 8 years) and healthy controls (21 females, 20 males; mean age 24.7 ± 6.7 years) (Yuan et al. 2018). For more details, see Table 2.

**Table 2:** Summary of human studies

Reference	Subjects: - Age - % Females	Aim, design and procedure	Number of subjects; duration of intervention	Groups + used substances - Dosage - Administration	Outcome and conclusions
Bahr et al. (2015a) (USA)	Cross-sectional group: - 9–15, 12.2 ± 2.5 yrs - 0%	Aim: evaluation of the impact of chronic and longitudinal RIS treatment on body weight and faecal microbiota composition Design: observational studies: cross-sectional and longitudinal Procedure: observation of the dynamics of body weight and gut microbiota alterations following the onset of RIS treatment. Cross-sectional group: stool sample after mean 3.6 ± 2.4 yrs of RIS treatment	Cross-sectional group: *N* = 18/at least 1-yr RIS treatment	Chronic RIS (*n* = 18) - NR - Per os No treated group (*n* = 10) - NA - NA	Metabolic: BMI *Z*-score increased by mean 0.31 ± 1.11 points over the course of treatment. Whereas in controls, it seemed to be unchanged (mean ΔBMI *Z*-score = 0.09 ± 0.61). PICRUSt analysis predicted KEGG orthologues: ↑ pathway levels for butyrate and propionate metabolism in RIS-treated group, ↑ SCFA production, alteration of tryptophan metabolism Gut microbiota: RIS treatment caused ↑ in Shannon diversity (5.9 vs 5.2), PcoA of unweighted UniFrac distances shown ↑ phylogenetic diversity, robust difference between the overall gut microbial profiles but no appreciable difference between significant vs no-significant BMI gain groups during treatment Bacterial abundances (RIS vs control): ↓ *Bacteroidetes/Firmicutes* ratio, ↑ phyla: Firmicutes*, *Proteobacteria*, and *Tenericutes*; families: Erysipelotrichaceae* and *Ruminococcaceae*; genera: Clostridium*, Lactobacillus*, Ralstonia*, and *Eubacterium.* (*more abundant in chronic RISP-treated children who had a significant gain in BMI compared to those with no BMI gain). ↑ Phylum *Actinobacteria*, *Tenericutes* order *Coriobacteriales* and species *Collinsella aerofaciens* in RISP group without weight gain (weight gain protective activity?) ↓ Phylum Bacteroidetes** and Verrucomicrobia**; genera: Prevotella** and *Alistipes*. (**less abundant in chronic RISP-treated children who had a significant gain in BMI compared to those with no BMI gain) Conclusion: Gut microbiota is altered in patients chronically treated with RIS and may be associated with weight gain and metabolic disturbances
	Longitudinal group: - 9–13, 11.7 ± 1.1 - 0% female Control group: - 10–14, 12.0 ± 1.8	Longitudinal group: stool sample within few days of starting treatment (mean 3.2 ± 5.2) and then monthly for 10 months. Control group for both groups: 10 to 14-year-olds psychiatrically ill but not treated with SGAs, only with psychostimulants or selective serotonin inhibitors (SSRIs)	Longitudinal group: *N* = 5 10-month RIS treatment	Longitudinal RIS (*n* = 5) - NR - Per os	Metabolic: BMI *Z*-scores increased by mean 0.28 ± 0.23 units. Gut microbiota: ↑ *Firmicutes/Bacteroidetes* ratio depending on the time of treatment. The percent abundance of *Firmicutes* and *Bacteroidetes* did not significantly correlate with the RIS-induced weight gain. Conclusion: Changes of gut microbiota composition starts few months after RIS treatment and correlate with weight gain. Probably due to small group size this correlation is not significant.
Flowers et al. (2017) (USA)	Adults with BD: - 46.0 ± 12.0 yrs - 69.6% No SGA treatment: - 51.7 ± 13.5 yrs - 73.9%	Aim: detection significant clustering of microbial communities between two groups of bipolar disorder patients (treated vs not treated with SGAs). Design: observational, cross-sectional. Procedure: study group: treated with SGAs (clozapine, olanzapine, risperidone, quetiapine, asenipine, ziprasodone, lurasidone, aripiprazole, paliperidone, and iloperidone) and some with antidepressants, mood stabilizers, lithium, benzodiazepines. Control group: no SGAs tx, but some were treated with the same drugs as the study group.	*N* = 117/use of SGAs at the time of stool collection	SGAs (*n* = 49) - NR - NR No SGA (*n* = 68) - NR - NR	SGAs vs No SGAs: Metabolic: ↑ BMI 31 ± 7 vs 27.5 ± 6 (significant after correcting for age and gender). Gut microbiota: ↓ Simpson diversity in females, ↑ family *Lachnospiraceae* in the whole cohort of patients treated with SGAs and cohort of obese subjects, no significant changes were observed in subgroup of obese and not obese patients despite on treatment); ↓ genera: *Akkermansia* (the whole cohort of patients treated with SGAs, cohort of obese subjects, subgroup of non-obese patients treated with SGAs), *Sutterella.* Conclusion: SGA treatment is associated with weight gain, decreased species richness in females and specific gut microbiota changes (which can play difficult to explain role in a weight gain process).
Yuan et al. (2018)	Adults with SCZ - 23.1 ± 8.0 yrs - 43.9%	Aim: to assess influence of RIS treatment on the metabolic parameters, redox system, inflammation relative to microbiota composition Design: observational, longitudinal. Procedure: the dynamics of metabolic outcome and gut microbiota alterations during 24 wks of RIS treatment (4 time points: baseline, 6, 12, 24 wks)	*N* = 82, 24 wks	RIS (*n* = 41) - Titrated from 1 mg/day up to 4–6 mg/day as clinically established - NR	Metabolic: ↑ weight (since 12 wks), ↑ BMI (since 6 wks); ↑ fasting serum glucose level (since 6 wks), ↑ HOMA-IR (since 6 wks); ↑ LDL (since 24 wks), ↑ triglycerides (since 12 wks); ↑ SOD (since 6 wks); ↑ serum levels of hs-CRP (since 12 wks), compared to baseline. At the endpoint serum levels of SOD negatively correlated with serum levels of LDL and HOMA-IR after controlling for potential confounding variables. Gut microbiota: ↓ *Clostridium coccoides* group (since 6 wks); ↑ *Bifidobacterium* spp. (since 6 wks), ↑ *Escherichia coli*; (since 6 wks), ↓ *Lactobacillus* spp. (since 12 wks) in comparison to baseline values. At baseline, *Bifidobacterium* spp. count negatively correlated with serum levels of LDL and *Escherichia coli* count negatively correlated with serum levels of triglycerides and hs-CRP (after controlling for age, gender, smoking status, and disease duration). In the hierarchical multiple linear regression model (adjusted for age, gender, smoking status, and disease duration), the changes in faecal *Bifidobacterium* spp. significantly correlated with the changes in weight over 24 wks. Conclusion: Body weight increase in SCZ patients treated with RIS are associated with abnormalities in the microbiota composition and the dysbiosis might contribute to the regulation of inflammation and oxidative stress thus metabolic malfunctions.

*ABX* antibiotics, *BD* bipolar disorder, *BMI* body mass index, *d* days, *GOS* galactooligosaccharides, *Gr*. group, *HFD* high-fat diet, *HOMA-IR* homeostasis model assessment-estimated insulin resistance, *hs-CRP* high-sensitivity C-reactive protein, *KEGG* Kyoto Encyclopedia of Genes and Genomes, *NR* not reported, *OLZ* olanzapine, *PCoA* principal coordinate analysis, *PICRUSt* Phylogenetic Investigation of Communities by Reconstruction of Unobserved States, *RIS* risperidone, *SCFA* short-chain fatty acid, *SCZ* schizophrenia, *SGA* second-generation antipsychotic, *SOD* superoxide dismutase, *tRMR* total resting metabolic rate, *tx* treatment, *VEH* vehicle, *vs* versus, *wks* weeks, *wk* week, *yrs* years

#### Risk of bias

An analysis of the overall risk of bias in rodent studies was limited by restricted information being provided. Results were heterogeneous with randomization in three articles (60%) (Davey et al. 2012, 2013; Bahr et al. 2015b), and no information regarding potential conflicts of interest was reported in one article (20%) (Morgan et al. 2014). Other key study quality indicators were poor, and an unclear risk for most types of SYRCLE’s bias was identified (Fig. S2). The reporting quality of the human studies was low (Bahr et al. 2015a) (score 13; 40.62%) and moderate (score 17; 53.12%) (Flowers et al. 2017), but a study by Yuan et al. (Yuan et al. 2018) was found to be of relatively high quality (score 20; 62.5%). For details, see Supplementary Figs. S1 and S2.

### Microbiota evaluation

#### Rodents

Bacteria in stool were tested in five studies [four OLZ (Davey et al. 2012, 2013; Morgan et al. 2014; Kao et al. 2018) and one RIS study (Bahr et al. 2015b)] using widely applied 16S rRNA sequencing methods. In two OLZ studies (Davey et al. 2012; Morgan et al. 2014) information was provided regarding microbiota diversity. In one RIS study (Bahr et al. 2015b), the number of bacterial operational taxonomic units (OTUs) was reported. The abundance of bacterial phyla was analysed in two OLZ studies (Davey et al. 2012, 2013) and one RIS (Bahr et al. 2015b) study, whereas bacterial classes were studied in two OLZ studies (Morgan et al. 2014; Kao et al. 2018) and bacterial genera in one RIS study (Bahr et al. 2015b). Briefly, a skewed *Firmicutes/Bacteroidetes* ratio was the most frequent observation of our SR, secondary to OLZ (Davey et al. 2012, 2013) and RIS (Bahr et al. 2015b) treatment. Detailed data are presented in Table 1.

Reduced microbiome diversity was identified in 16 rats following OLZ treatment (both genders) (Davey et al. 2012) and 24 female mice after HFD and OLZ regimen (Morgan et al. 2014). Only one study reported data on fewer OTUs in female mice treated with RIS (Bahr et al. 2015b). OLZ treatment in rats increased the abundance of *Firmicutes* from 6.40 to 16.01% and decreased the abundance of *Bacteroidetes* from − 6.69 to − 4.30%. The effect was dose dependent and greater in females (Davey et al. 2012, 2013). In addition, the abundance of *Actinobacteria* (females 2 mg, − 3.38%; 4 mg, − 3.57%) and *Proteobacteria* (females 2 mg, − 1.45%; 4 mg, − 0.83%; males 2 mg, − 2.21%) was decreased compared with vehicle-treated rodents (Davey et al. 2012). The other OLZ rodent study found an increase in the relative abundance of classes *Erysipelotrichia* (up to 3.40%) and *Gammaproteobacteria* (up to 0.45%), whereas the abundance of class *Bacteroidia* was reduced (− 5.30%) (Morgan et al. 2014). Only in a single study (Kao et al. 2018) OLZ administration caused no variations within microbiota composition in comparison with vehicle-treated rodents, possibly because of the short treatment duration and the dose of the administered drugs. However, OLZ was administered prior to B-galactooligosaccharide (B-GOS) and attenuated prebiotic mode of action (↑ *Bifidobacterium*; ↓ *Escherichia/Shigella* spp., *Coprococcus* spp., *Oscillibacter* spp., *C. coccoides* spp., *Roseuria Intestinalis* cluster, and *Clostridium* XVIII cluster) which indirectly suggested that this SGA influenced gut microbiota. Also, acetate concentration in faeces, a by-product of gut microbiota, increased in OLZ-treated rodents, implying that microbiome structure and function could be at least partly changed by SGA (Kao et al. 2018).

Although RIS was implemented in six rodent experiments (from three articles), a microbiota analysis was performed in only one of them. An increase in the relative abundance of *Firmicutes* (32.6%) was found with a reciprocal decrease in the relative abundance of *Bacteroidetes* (− 22.40%) in drug-treated subjects (Bahr et al. 2015b). *Alistipes* spp. and *Lactobacillus* spp. were more prevalent in control-treated rodents, whereas the population of *Allobaculum* spp. increased (36.5%) in the RIS group (Bahr et al. 2015b). Furthermore, SGAs were shown to possess antibacterial properties in vitro; OLZ inhibited the growth of anaerobic bacteria (Bahr et al. 2015b), and diminished the growth of *Escherichia coli* NC101 but not *Enterococcus faecalis* OGIRF cultures (Morgan et al. 2014).

#### Humans

In two human studies (Bahr et al. 2015a; Flowers et al. 2017), the bacteria in stools were tested using 16S rRNA sequencing methods, while in a third study (Yuan et al. 2018), the copy numbers of five bacterial genera (*Bifidobacterium* spp., *Clostridium coccoides* group, *Lactobacillus* spp., and *Bacteroides* spp.) were determined by means of qPCR analysis. The difference in microbial communities between observed groups was calculated using principal coordinate analysis (PCoA). One study assessed Shannon diversity (all sample species) (Bahr et al. 2015a), and another focused on Simpson (dominant species) diversity (Flowers et al. 2017). The abundance of bacterial phyla was analysed in one study (Bahr et al. 2015a), whereas bacterial families were analysed in two human studies (Bahr et al. 2015a; Flowers et al. 2017) and bacterial genera in all human studies (Bahr et al. 2015a; Flowers et al. 2017; Yuan et al. 2018). The *Firmicutes/Bacteroidetes* ratio was reported in only the RIS study (Bahr et al. 2015a).

Flowers et al. (2017) reported reduced Simpson diversity in females treated with SGAs which remained significant after adjusting for age, BMI, and benzodiazepine treatment (*p* = 0.002, *β* = − 4.6, *R*^2^ = 0.12). A greater abundance of *Lachnospiraceae* was observed in obese patients treated with SGAs, whereas *Akkermansia* and *Sutterella* abundance was higher in controls, though only the first two differences (*Lachnospiraceae* and *Akkermansia*) remained significant (*p* = 0.001 and *p* = 0.03) after adjusting for BMI and gender. Lastly, the study found that *Akkermansia* were less prevalent in non-obese SGA users (*p* = 0.005) (Flowers et al. 2017).

Bahr et al. (2015a) identified a significantly higher Shannon diversity index (0.7 points) and phylogenetic diversity in 18 male adolescents chronically (> 12 months) treated with RIS compared to psychiatric control participants. The *Bacteroidetes/Firmicutes* ratio was significantly lowered (0.15 vs 1.24, respectively, *p* < 0.05) in chronic and short-term (1–3 months) RIS users. The tendency to decrease the *Bacteroidetes/Firmicutes* ratio observed in short-term RIS users seemed to correlate with the change in BMI *Z*-score, which is a function of both age and gender and shows the deviation from the population mean. The observed results were not significant, probably because of the small number of patients. Moreover, the authors observed that long-term treatment with RIS and significant weight gain in RIS users were associated with alterations in the gut microbiome: an increased abundance of the phylum *Proteobacteria*, families *Erysipelotrichaceae* and *Ruminococcaceae*, and genera *Clostridium*, *Lactobacillus*, *Ralstonia*, and *Eubacterium* and decreased abundance of the genera *Prevotella* and *Alistipes*. Interestingly, the abundance of phylum *Actinobacteria* and species *Collinsella aerofaciensin* was elevated in the RIS group without weight gain, which suggests a protective effect of these bacteria in chronic RIS users (Bahr et al. 2015a).

In a study by Yuan et al. (2018), authors found that 24-week RIS treatment was associated with a significant overall increase in the copy numbers of faecal *Bifidobacterium* spp. (*F*_(3,160)_ = 7.298, *p* < 0.001; week 0, 6.72 ± 1.35 l g copies/g; week 24, 7.24 ± 0.78 l g copies/g) and *Escherichia coli* (*F*_(3,160)_ = 8.280, *p* < 0.001; week 0, 7.58 ± 0.68 l g copies/g; week 24, 8.03 ± 0.66 l g copies/g). Interestingly, the copy numbers of faecal *Bacteroides* spp. did not change over 24 weeks of RIS treatment (*F*_(3,160)_ = 2.188, *p* = 0.092). They also noticed that after 6 weeks of treatment, the copy numbers of *Bifidobacterium* spp. (at 6 weeks *p* < 0.05, 12, 24 weeks *p* < 0.001) and *Escherichia coli* (at 6 weeks *p* < 0.05, 12 *p* < 0.01, 24 *p* < 0.001) elevated. Copy numbers of *Clostridium coccoides* group were lower after 6 weeks of treatment (at 6 and 12 weeks *p* < 0.01, 24 weeks *p* < 0.001), and *Lactobacillus* spp. was decreased at 12 and 24 weeks of RIS administration (*p* < 0.001).

### Metabolic outcome

#### Rodents

The effect of OLZ administration on body weight was measured using different experimental models in four studies (experiments = 5) including C57BL/6J female mice (experiments = 2) (Morgan et al. 2014) or both genders of Sprague–Dawley rats (experiments = 3) (Davey et al. 2012, 2013; Kao et al. 2018). The impact of RIS on body weight was determined in three studies (experiments = 6); one study included C57BL/6J female mice (Bahr et al. 2015b), and two other studies (conference abstracts) did not report mouse gender (Grobe et al. 2015; Riedl et al. 2017). We did not conduct aggregated analysis concerning body weight, due to methodological differences and various ways of expressing measured values.

In general, administration of OLZ increased body weights in female rats (Davey et al. 2013; Kao et al. 2018) and female mice (Morgan et al. 2014). This effect was dose independent in two studies (Davey et al. 2012, 2013). In one study, OLZ administration caused increased adiposity (percentage of body fat), even after correction for weight gain (Morgan et al. 2014). The body weight increases induced by OLZ was counteracted by antibiotic administration (Davey et al. 2013) and lack of bacteria (germ-free mouse model), and potentiated by an HFD (Morgan et al. 2014). The increase in body weight induced by RIS administration was not affected by antibiotics (Bahr et al. 2015b). In rodents receiving RIS, the non-aerobic resting metabolic rate (RMR) was suppressed in mice in three studies (Grobe et al. 2015; Bahr et al. 2015b; Riedl et al. 2017). One study reported no direct information regarding body weight but identified that, in RIS-treated rodents, the suppression of tRMR was not affected by cecectomy (Riedl et al. 2017). Increased fat mass and free fatty acid release and elevated expression of lipogenic enzymes were observed in 12 female rats (Davey et al. 2013). Importantly, in six female rats, administration of SGAs resulted in elevated expression of macrophage marker CD68 in adipose tissue, indicating that body weight gains were associated with recruitment of macrophages into the fat mass (Davey et al. 2013).

#### Humans

In 18 male children chronically treated with RIS, the BMI *Z*-score increased by a mean 0.31 ± 1.11 points, and the BMI *Z*-score increased over 10 months of treatment (mean 0.28 ± 0.23 units) in a longitudinal study arm (Bahr et al. 2015a). Flowers et al. (2017) observed higher BMI in patients receiving SGAs (31 ± 7 vs 27.5 ± 6, *p* = 0.006; after correcting for age and gender, *p* = 0.04). Yuan et al. (2018) discovered that RIS treatment caused a significant increase in weight (*F*_(3,160)_ = 4.331, *p* = 0.006), BMI (*F*_(3,160)_ = 5.025, *p* = 0.002), fasting serum glucose levels (*F*_(3,160)_ = 5.081, *p* = 0.002), triglycerides (*F*_(3,160)_ = 3.428, *p* = 0.019), LDL (*F*_(3,160)_ = 3.973, *p* = 0.009), and HOMA-IR (*F*_(3,160)_ = 10.187, *p* < 0.001). At a week 6 of treatment, increases in BMI (week 0, 20.54 ± 4.87 kg/m^2^; week 6 21.96 ± 5.36 kg/m^2^; *p* < 0.05), glucose (week 0, 4.37 ± 1.03 mmol/l; week 6, 4.63 ± 0.81 mmol/l; < 0.01), HOMA-IR (week 0, 0.97 ± 0.67; week 6, 1.39 ± 1.17; *p* < 0.001), and LDL (week 0, 2.22 ± 1.25 mmol/l; week 6, 2.62 ± 1.53 mmol/l; *p* < 0.05) were observed. At 12 and 24 weeks, all metabolic parameters mentioned above were also significantly increased (BMI—week 12, 22.54 ± 5.7 kg/m^2^; *p* < 0.01; week 24, 22.88 ± 6.97 kg/m^2^; *p* < 0.001; LDL—week 12, 2.69 ± 1.36 mmol/l; *p* < 0.01; week 24, 2.63 ± 1.19 mmol/l; *p* < 0.01). Additionally, weight increased significantly (week 12, 63.49 ± 18.94 kg, *p* < 0.01; week 24, 62.85 ± 19.73, *p* < 0.01), as well as serum triglyceride level (week 0, 0.96 ± 1.33 mmol/l; week 12, 1.28 ± 0.97 mmol/l, *p* < 0.01; week 24, 1.37 ± 1.37 mmol/l; *p* < 0.001).

No other metabolic investigations were undertaken; however, Bahr et al. (2015a) performed Phylogenetic Investigation of Communities by Reconstruction of Unobserved States (PICRUSt) analyses and found that bacterial orthologues enriched in chronic RIS patients compared to controls were responsible for environmental information processing pathways and cellular processes, including short-chain fatty acid and tryptophan metabolism. In persons free of psychiatric treatment, there were more orthologues involved in bacterial metabolic pathways, such as vitamin metabolism. Detailed data extracted from original papers included in our systematic review are provided in Table 2.

### The role of microbiota in metabolic outcomes

#### Rodents

Most studies investigating this hypothesis have been performed in different experimental models. Morgan et al. (2014), in their germ-free experiment in gnotobiotic mice, showed that the microbiota is necessary to induce metabolic changes after OLZ treatment. One experiment assumed that the relative abundance of class *Erysipelotrichi*, which is increased by OLZ, is linked to rapid weight gain; every 1% increase in abundance resulted in a weight gain of 0.7%. The same pattern, though more pronounced, was identified relative to the class *Actinobacteria* (for which an OLZ effect was not observed); every 1% increase in abundance resulted in a weight gain of almost 5% (Morgan et al. 2014). In a study by Kao et al. (2018) 2-week intraperitoneal OLZ administration in female rodents elevated body weights, and prebiotic therapy attenuated this effect. The study indicated no OLZ-induced alterations of gut microbiota which means that B-GOS supplementation may prevent weight gain independently of its influence on gut microbiota. In a rodent RIS intervention (Bahr et al. 2015b), the medication caused a significant increase in weight (2.8 g) compared to control mice, and co-administration of antibiotics had no significant effect on the weight gain. When performing microbiota transfer from RIS-treated mice, recipients had a 16% reduction in total resting metabolic rate (tRMR) due to a reduction in non-aerobic RMR. tRMR states for the largest portion of total energy need thus is relevant to describe metabolic outcomes (Astrup et al. 1999) Similarly, transfer of the phageome from RIS mice resulted in a significant weight gain in recipients relative to the vehicle study arm (*p* < 0.05) (37). Two studies obtained indirect data on the metabolic influence of microbiota changes following RIS treatment (Grobe et al. 2015; Riedl et al. 2017). In the study by Grobe et al. (2015), faecal transplants from RIS-treated rodents resulted in elevated body mass through non-aerobic RMR suppression, which was found to be unaffected by cecectomy (Riedl et al. 2017). Study conclusions are shown in Table 3.

**Table 3:** Summary of the rodent studies showing a relationship between metabolic changes and microbiota alteration after SGA treatment

Study	SGA	Relationship	Comment
Davey et al. (2012)), Ireland	OLZ	Weak	Metabolic disturbances, inflammation, and microbiota alterations were observed only in female mice. In males, impact on microbiota and metabolism was minimal.
Davey et al. (2013), Ireland	OLZ	Strong	Metabolic effects of OLZ were associated with gut microbiota changes and were attenuated by antibiotics, which strongly reduced gut microbiota content.
Morgan et al. (2014), USA	OLZ	Strong	Results of few experiments shown that gut microbiota was necessary to induce weight gain (germ-free mice model) and that weight gain was related to the relative abundance of the special bacteria (cross-over study design).
Kao et al. (2018), UK	OLZ	Not observed	Short-term OLZ treatment did not affect faecal bacterial composition in female rats.
Bahr et al. (2015a), USA	RIS	Strong	Faecal and phage transplantation from mice treated with RIS caused weight gain and decreased energy expenditure.
Grobe et al. (2015), USA	RIS	Strong	Faecal transplantation from mice treated with RIS caused decreased tRMR.
Riedl et al. (2017), USA	RIS	Not clear	Cecectomy does not affect decreased tRMR after RIS treatment. It means that antibacterial properties of RIS are enough to reduce tRMR, and a further reduction of bacteria count via cecectomy is not required or that the mechanism does not depend on intestinal microbiota.

#### Humans

In humans, the data are less clear. Bahr et al. found increased BMI *Z*-scores in humans treated with RIS (mean 0.31 ± 1.11 points over the treatment course), whereas in controls, the scores seemed to be unchanged (Bahr et al. 2015a). Chronic treatment with RIS and a significant gain in BMI resulted in a lower *Bacteroidetes*/*Firmicutes* ratio compared with the control group. Moreover, differences in bacterial composition were observed in RIS-treated children who had BMI gains compared to those who did not. Detailed data concerning the association of differences in microbiota abundance depending on body weight gain are presented in Table 2. In the initial phase of RIS treatment lasting 10 months, BMI *Z*-scores increased a mean 0.28 ± 0.23 units, which seemed to correlate with a decreased *Bacteroidetes/Firmicutes* ratio starting 1–3 months after treatment initiation (the observed result was not significant, likely due to the small sample size).

In the second human study, SGA treatment resulted in higher BMIs, even after adjusting for patient age and gender followed by significant elevation in the abundance of *Lachnospiraceae*. *Akkermansia* counts were significantly lowered in the SGA-treated group, including non-obese patients. Surprisingly higher *Lachnospiraceae* and lower *Akkermansia* counts were observed in non-SGA-treated obese individuals (Flowers et al. 2017).

In the last human study, the authors (Yuan et al. 2018) found that at baseline *Bifidobacterium* spp., counts negatively correlated with serum levels of LDL and *Escherichia coli* count was negatively correlated with serum levels of triglycerides and hs-CRP, even after controlling for age, gender, smoking status, and disease duration. Following the treatment, a decrease in *Clostridium coccoides* group (since 6 weeks) and *Lactobacillus* spp. (since 12 weeks) and elevations in numbers of *Bifidobacterium* spp. (since 6 weeks) and *Escherichia coli* (since 6 weeks), in comparison to baseline values, were reported. When they conducted hierarchical multiple linear regression, only the differences in faecal *Bifidobacterium* spp. count significantly correlated with the weight changes over 24 weeks of RIS treatment (Yuan et al. 2018). A summary of the evidence from human studies is provided in Table 4.

**Table 4:** Summary of the human studies showing a relationship between metabolic changes and microbiota alteration after SGA treatment

Study	SGA	Relationship	Comment
Bahr et al. (2015a), USA	OLZ	Moderate	A specific microbiota alteration is observed in weight-gained children chronically treated with RIS. Longitudinal study showed correlation between changes in gut microbiota and weight gain caused by RIS treatment (result was not significant probably due to small sample size).
Flowers et al. (2017), USA	Different SGAs	Weak	Changes of a specific bacteria abundance are associated with lack of weight gain after SGA treatment, but results are difficult to explain.
Yuan et al. (2018), China	RIS	Strong	Weight gain in patients treated with RIS is significantly correlated with faecal bacterial abundance.

## Discussion

**Fig. 2:**
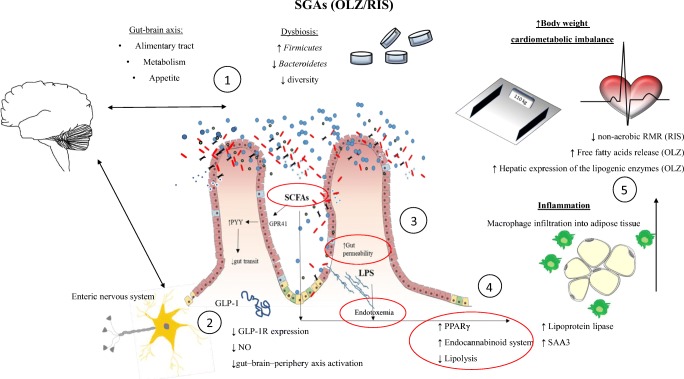
Schematic presentation of possible mechanisms of metabolic disturbances secondary to SGA treatment. SGAs affect the gut microbiota, causing shifts in two major phyla: *Firmicutes* and *Bacteroidetes*. (1) The gut-brain axis controls metabolism, appetite, and digestive tract functions and may become altered under dysbiosis insult. (2) SCFAs activate G protein binding receptor (GPR) which is followed by secretion of YY peptide (PYY) resulting in lowered gut motility. Dysbiosis also induces GLP-1 resistance, followed by diminished GLP-1 receptor expression and hampered nitric oxide production. Consequently, the gut-brain peripheral axis responsible for insulin secretion and stomach emptying is inhibited. (3) Dysbiosis is associated with loss of integrity of the gastrointestinal barrier and increased permeability of the intestinal mucosa for gut lumen antigens, including bacterial LPS. (4) High-energy SCFAs and endotoxemia affect multiple metabolic pathways. Activation of the differentiation of peroxisomal gamma proliferator-activated receptors (PPARγ) and the pro-inflammatory endocannabinoid system takes place which regulates fatty acid synthase (FAS), enhancing hepatic de novo lipogenesis. LPS exacerbates hepatic steatosis and insulin resistance. Consequently, macrophages infiltrate adipose tissue and (5) body weight increases. The mechanisms include suppression of non-aerobic RMR (OLZ), increased free fatty acid release, and elevated hepatic expression of the lipogenic enzyme fatty acid synthase (RIS). Possible targets counteracted by prebiotics and probiotics are circled in red. FAS fatty acid synthase, GLP-1 glucagon-like peptide 1, GPR G protein binding receptor, LPS lypopolysaccharide, NO nitroic oxide, OLZ olanzapine, PPARγ peroxisomal gamma proliferator-activated receptors, PYY YY peptide, RIS risperidone, RMR resting metabolic rate, SAA3 serum amyloid A3, SCFAs short-chain fatty acids

To the best of our knowledge, this is the first SR investigating the effect of SGAs on intestinal microbiota in relation to frequent metabolic adverse events associated with their use in clinical practice. This SR aimed to find answers to three questions: (1) do SGAs affect intestinal microbiota resulting in dysbiosis, (2) whether SGA-related metabolic disorders are associated with dysbiosis, and finally (3) to elucidate the mechanisms behind SGA treatment and dysbiosis leading to body weight and metabolic disturbances (Fig. 2) (Delzenne et al. 2011). Although numbers of existing rodent and human studies are limited, we found that dysbiosis secondary to SGA treatment can play a role in metabolic alterations, including weight gain.

The human gut is colonized by roughly 39 × 10^9^ bacterial cells (Abbott 2016), with other species, including *Archaea*, eukaryotes, fungi, and viruses (Consortium et al. 2012). Dysbiosis is an imbalance in the number, composition, or function of bacteria in a given environment. Dysbiosis has been confirmed in all but one of the experimental studies in which the content of bacteria in stools was assessed. The most frequent observation was an increase in the *Firmicutes/Bacteroidetes* phyla ratio. It should be emphasized that the ratio of *Firmicutes/Bacteroidetes* was elevated in all experimental studies investigating this parameter. These two phyla are generally dominant in the human intestinal microbiome, comprising approximately 90% of the gut microbiota (Consortium et al. 2012). The *Bacteroidetes* phylum has been found to synthesize acetate and propionate, while *Firmicutes* mainly by-product is butyrate (den Besten et al. 2013). Beneficial effects of SCFAs were shown as far as gastrointestinal functions, neuro/immune regulation, and host metabolism were concerned (Maciejewska et al. 2018; van de Wouw et al. 2018). Proper concentration of SCFAs acting via its receptors is crucial for energy homeostasis. When *GPR*43-deficient mice were fed with normal diet, they started to accumulate fat and SCFA-dependent activation of the receptor resulted in suppressed insulin signalling within the adipose tissue thus inhibited fat storage (Kimura et al. 2013). In 2005, Ley et al. hypothesized that differences in gut microbial ecology may be an important factor affecting energy homeostasis (Ley et al. 2005). Later, an increased *Firmicutes/Bacteroidetes* ratio was observed in obese rodents and humans (Turnbaugh et al. 2009).

Elevated gut microbiota fermentative metabolism by over-represented *Firmicutes* may therefore promote more intensive intestinal monosaccharide absorption, energy extraction from non-digestible food components, hepatic de novo lipogenesis, and adipocyte fatty acid storage. The analysis of the SCFA concentration represents an indirect way to analyse microbiota composition (at least a skewed *Firmicutes* count) and can be viewed as a marker of microbiota metabolic function.

Observations from experimental studies have only been partially confirmed in human studies. Bahr et al. (2015a) observed increased *Firmicutes/Bacteroidetes* ratios in both cross-sectional and longitudinal studies. In other human studies, bacterial phyla were not reported. Another result of experimental studies confirmed in the human trial was the increase in the abundance of class *Erysipelotrichi*, which was found to be related to the occurrence of non-alcoholic fatty liver disease (Spencer et al. 2011; Henao-Mejia et al. 2012; Raman et al. 2013). Of note, as recently demonstrated by Schwarz et al. (2018) in first-episode psychosis patients, the abundance of predominantly *Lactobacillus* from *Firmicutes* phyla was increased which correlated negatively with different clinical scores of schizophrenia. Also, after 12 months of treatment in patients with smaller alterations within gut microbiota at baseline, remission rate was more frequent. However, patients were not drug-naïve, and received antipsychotics for approximately 20 days, which at least partly confirm the higher abundance of *Firmicutes* following SGA treatment. We found no more data on how SGA-induced dysbiosis may affect the clinical course of the disease and consequently treatment success. To close the circle, *Lactobacillus* represents only a single genus within the *Firmicutes* phyla; thus, the effect of SGAs on *Firmicutes/Bacteroidetes* ratio in humans could only be speculative and requires further research.

The mechanism of dysbiosis secondary to SGAs has not been fully explained. In two studies included in the present SR, both OLZ (Morgan et al. 2014) and RIS (Bahr et al. 2015b) had an antimicrobial nature. Moreover, the principal coordinate analysis of the uniweighted UniFrac distance showed that antibiotics had a synergistic influence on gut microbiota with RIS. This effect is predominantly typical of drugs subject to enterohepatic circulation and intensely excreted in the bile (Morgan et al. 2014). Bactericidal activity causes dysbiosis via elimination of specific bacteria from the gastrointestinal tract. The observed phenomenon of a greater increase in body mass in naive SGA users (Maayan and Correll 2010) can be caused, among other reasons, by the induction of bacterial resistance in relation to the repeated use of SGAs. It should be emphasized that the observed results are not unambiguous and easy to interpret. The composition of intestinal bacteria varies among individuals and is analogous to fingerprints. Also, in individual studies (also experimental), various taxonomic groups of bacteria were analysed, and their content was analysed only in stools. The composition of bacteria in the stool is more stable and does not depend on external factors compared with the composition of bacteria in the small intestine. Changes in the microbiota of the small intestine have a much greater effect on the metabolic functions of the body. Therefore, in further experimental studies, attention should be paid to this problem.

To address the question of whether SGA-induced dysbiosis may be responsible for metabolic malfunctions, we analysed a few experimental models. Two studies introduced antibiotic cocktails as experimental variables (Davey et al. 2013; Bahr et al. 2015b). Co-administration of antibiotics, which significantly reduced gut bacterial content, prevented dysbiosis and its metabolic consequences. On the other hand, antibiotics used only to slightly modify gut microbiota had antibacterial activity similar to SGAs but did not influence their metabolic effects. These observations confirm a potential causal relationship between dysbiosis caused by the intake of SGAs and metabolic disorders. Similar results were previously reported in mouse models (Mathur et al. 2016). As some antipsychotics have been documented to possess antimicrobial activity (Kristiansen 1979), the administration of these drugs may resemble the mode of action of low-dose antibiotic cocktails, which may be responsible for increased body mass as observed in livestock (Morgan et al. 2014). In contrast, some non-absorbable antibiotics (e.g., rifaximin) have been shown to reduce the abundance of methanogenic bacteria (Mathur et al. 2016), resulting in significant weight loss in obese individuals with diabetes (Riedl et al. 2017). The antibiotic effect on body fat composition is plausibly dose- and age-related (Cox et al. 2014). Additionally, Morgan et al. (2014) implemented HFD, which induces changes in the composition of the gut microbiota toward an obesogenic composition with subsequent metabolic consequences, including metabolic syndrome (Yang et al. 2017). However, HFD had no impact on the anthropometric indices of germ-free mice (Bäckhed et al. 2007), which may indicate that metabolic disturbances during OLZ treatment are sourced from altered gut microbiota. It was observed that both HFD and OLZ have a synergistic effect on gut microbiota composition but weight gain in mice receiving OLZ is more rapid than feeding only with HFD. This means that in the case of metabolic disorders caused by OLZ administration, in addition to the SGA-mediated obesogenic effect on gut microbiota, other factors should also be taken into consideration. Also, it is possible that such treatment is more pronounced in comparison to HFD alone. Morgan et al. (2014) confirmed the relationship between OLZ-induced dysbiosis and metabolic disorders using a germ-free model, in which they showed that the lack of bacteria in the gastrointestinal tract in mice receiving OLZ did not cause weight gain, and their conventional housing leading to the colonization of the digestive tract resulted in induction of weight gain. A similar observation was made by Bäckhed et al. (2007) who found that gnotobiotic mice had less body fat than mice housed under conventional conditions. Morgan et al. (2014) confirmed the relationship between weight gain and OLZ administration using the cross-over model, proving that the relative abundance of bacteria of the *Erysipelotrichi* class was related to weight gain.

Another argument for the relationship between the occurrence of dysbiosis and metabolic disorders with an increase in body weight caused by the administration of OLZ was provided by Davey et al. (2012) who observed microbiota and metabolic disorders only in female mice. In male mice, metabolic side effects and impact on bacterial abundance were minimal. In human trials included into present SR, no differences in metabolic outcome between males and females were reported (Flowers et al. 2017; Yuan et al. 2018). However, such gender-dependent discrepancies were reported earlier and may be due to drug pharmacokinetic differences (Harris et al. 1995; Beierle et al. 1999) which further support the necessity to conduct more studies. Bahr et al. and Grobe et al. provided the strongest evidence for a link between the occurrence of metabolic disorders and gut microbiota (Grobe et al. 2015; Bahr et al. 2015b). The authors observed that faecal and phage transplantation from mice treated with RIS caused weight gain and decreased rest metabolic rate. The phenotypic effect of faecal transplantation is considered as a very strong evidence of intestinal microbiota, also in terms of its effect on metabolism. For example, lean mice-derived microbiota transferred to germ-free mice resulted in lower body fat increases than the transfer of the ob/ob mice microbiome (Turnbaugh et al. 2006). Riedl et al. did not confirm this observation and found that cecectomy (associated with marked reduction of gut microbiota counts) did not influence the suppression of non-aerobic RMR caused by RIS treatment (Riedl et al. 2017). This observation may indicate that RIS is an antibacterial agent that reduces RMR, and a further reduction of bacteria count via cecectomy is not needed. However, it is also possible that the mechanism does not depend on intestinal microbiota. It should be emphasized that the results of this study come from a conference summary, and may not contain the necessary data for accurate interpretation of these observations (Riedl et al. 2017).

Human studies provide little evidence on the relationship between metabolic changes and microbiota alterations after SGA treatment. Bahr et al. (2015a) found specific microbiota alterations in weight gain of children chronically treated with RIS, although the results were not significant probably due to the small sample size. However, PcoA of unweighted UniFrac distances showed elevated phylogenetic diversity and robust differences between the overall gut microbial profiles, but no appreciable differences between significant versus non-significant BMI gain groups during treatment. Bioinformatic analysis (Bahr et al. 2015a) demonstrated increased butyrate and propionate metabolism in the RIS-treated group, as well as SCFA production and impairments in tryptophan metabolism. Flowers et al. (2017) found that low abundance of bacteria from the genera *Akkermansia* was associated with a lack of weight gain after SGA treatment. However, these results are difficult to explain. *Akkermansia muciniphila* may serve as a negative marker of inflammation as it was found that the abundance of this genus is reduced under the regimen of HFD and its decline correlated negatively with lipid synthesis, plasma markers of insulin resistance, cardiovascular risk, and adiposity in rodents (Schneeberger et al. 2015). In a recent study published by Yuan et al. (2018), it was concluded that weight gain in patients treated with RIS was significantly correlated with an increase of faecal *Bifidobacterium* spp. abundance. *Bifidobacterium* spp. have an anti-inflammatory effect against systemic inflammation, and an increased abundance could be a compensatory reaction after weight gain and inflammation.

To answer the question whether dysbiosis caused by SGA administration is related to the occurrence of metabolic disorders, prospective clinical trials, and further experimental studies in which the same taxonomic groups of bacteria and their metabolic functions are assessed are necessary.

Based on the results of the analysed studies, we attempted to describe the mechanism of metabolic disorders originating from SGA treatment. Based on the current systematic review, we conclude that inflammation is critical to inducing weight gain and other metabolic alterations secondary to SGA use (Straczkowski et al. 2002; Kim et al. 2006). First, dysbiosis affects energy homeostasis of the body and lipid metabolism (Slyepchenko et al. 2016; Boulangé et al. 2016). Also, dysbiosis alters the structure and function of the intestinal barrier and may cause the translocation of bacterial antigens into the systemic circulation (Küme et al. 2017). Data on the presence of various microorganisms in extracolonic tissues and organs (Nagpal and Yadav 2017) following HFD (Wirostko et al. 1990) in obese individuals are increasing (Schwiertz et al. 2010). Bacterial lipopolysaccharide (LPS) components of gram-negative bacteria and cyanobacteria and high-energy SCFAs play major roles in energy harvesting. These molecules, among others, activate the G protein binding receptor (GPR), followed by secretion of the YY peptide (PYY), resulting in decreased intestinal motility, increased fat storage by reduced expression of the lipoprotein lipase inhibitor (fasting-induced adipose factor (FIAF)), activation of the differentiation of peroxisomal gamma proliferator-activated receptors (PPARγ) and the pro-inflammatory endocannabinoid system, respectively, and the development of adipose mass. These components regulate fatty acid synthase (FAS), enhancing hepatic de novo lipogenesis. LPS exacerbates hepatic steatosis and insulin resistance (Marlicz et al. 2014; Jin et al. 2017; Rorato et al. 2017). Macrophages possess the ability to phagocytose LPS, migrate to peripheral tissues, and release pro-inflammatory cytokines. Consequently, adipokine synthesis is decreased and leptin and ghrelin levels increase. All of these pathways sustain systemic inflammation (Tilg and Kaser 2009, 2011; Park and Scherer 2011) and may contribute to metabolic alterations in patients exposed to HFD with type 2 diabetes mellitus (Burcelin 2012). Unfortunately, LPS and gut barrier function were not measured in the analysed studies.

In immune-related pathogenesis of obesity, bacterial LPS and peptidoglycans, as pro-inflammatory agents, activate pathogen recognition receptors (PRRs) on macrophages and neutrophils, and as part of the non-specific immune response (Burcelin 2012) are responsible for hyperinsulinaemia and insulin resistance (Saberi et al. 2009). Furthermore, immune-related mechanisms may contribute to gut microbial alterations. For example, mice lacking *TLR5* develop dysbiosis followed by metabolic syndrome (Tilg and Kaser 2009; Tremaroli and Bäckhed 2012). Obesogenic-type dysbiosis induces inflammation within the gut and affects neurotransmitter levels and the gut-brain axis function (Collins et al. 2012). Skewed production of serotonin in the gut (Clarke et al. 2013) may be at least partly responsible for weight gain secondary to SGA treatment via microbial alterations (Collins et al. 2012). However, none of the studies included in this SR reported such an association. A study by Kao et al. (2018), however, demonstrated that the B-GOS mode of action is independent of the serotonin pathway.

Only one study in this systematic review reported elevated levels of TNF-α when co-administered with B-GOS (Kao et al. 2018). TNF-α was found to act as weight gain suppressant and influence adipocytes lipid metabolism (Langhans and Hrupka 1999; Coppack 2001). Unexpectedly, another study discovered macrophage infiltration of adipose tissue (Davey et al. 2013). However, Xu et al. (2003) discovered that macrophage infiltration of adipose tissue is associated with the development of inflammation and insulin resistance in obese individuals. In one human study (Yuan et al. 2018), elevated concentrations of hs-CRP and decreased levels of SOD in patients with SCZ were found, and these alterations were more pronounced following RIS treatment, proving that both oxidative stress and inflammation may be responsible for metabolic malfunctions. No other research evaluated the role of gut permeability in systemic inflammation. Based on data included in this systematic review, we postulate that the assessment of intestinal permeability may serve as a surrogate marker of both gut dysbiosis and metabolic alterations. This should be verified in well-controlled trials in obese individuals. However, Davey et al. (2012) have shown that OLZ administration was associated with an inflammation in female mice that can suggest that intrinsic properties of this agent may directly alter inflammatory mechanisms.

We conclude that metabolic disturbances during SGA treatment may be the consequence, at least in part, of gut dysbiosis. Numerous trials confirmed a beneficial effect of prebiotics and probiotics on gut microbiota composition, with a lower risk of metabolic and weight disturbances. We found only one study in which B-GOS administration attenuated OLZ-mediated weight gain independently of serotonin pathways and acted positively on gut microbiota composition when utilized alone. Therefore, we suggest further research, considering probiotic/prebiotic/synbiotic therapy with concomitant SGA treatment. Such co-therapy may not only positively prevent or reduce weight gain but also modulate fasting glucose and glycated haemoglobin, dyslipidaemia, total and LDL cholesterol, and hypertension. Moreover, as discovered by Kao et al. (2018), prebiotics may elevate cortical glutamate receptor subunit mRNA expression (GluN1) in contrast to reductions of this receptor density typically seen in chronic SGAs users affecting their cognition (Krzystanek et al. 2015, 2016) negatively. Of particular interest is a search for target probiotic strains, such as *Collinsella aerofaciens*, which is increased in children treated with RIS without commensurate weight gains (Bahr et al. 2015a). Potential next-generation probiotic bacteria include *Akkermansia*, *Bacteroides* spp., and *Eubacteriumhalli*, as well as bacterial structural elements (cell wall proteins) and metabolites (e.g., SCFAs) (Romaní-Pérez et al. 2017; Muszyńska et al. 2018)

This systematic review has at least four limitations. First, the number of studies identified and included in this review was low. Most of the included studies were conducted in rodent models with an unclear risk of bias. Thus, the results of these studies may not be fully extrapolated to humans. Second, although reliable molecular techniques were used for microbiota analyses in all of the included studies, none of the research used intestinal samples. Therefore, it is impossible to conclude the microbiota composition in various parts of the gastrointestinal tract. Third, none of the human studies were randomized and placebo controlled. Notably, SGAs have also been found to affect food intake habits (Reynolds and McGowan 2017) and, consequently, microbiota composition (Turnbaugh 2017). Although such a relationship was demonstrated in one study, and in female rodents only (Davey et al. 2012), detailed observations should be the subject of further research. Thus, our conclusions need to be cautiously considered.

In conclusion, this systematic review proves that alterations in the gut microbiota composition causing low-level inflammation and decreased energy expenditure can play a role in body weight gain during SGA treatment. Experimental research targeting the gastrointestinal microbiota to discover the exact mechanism of SGA action associated with poor metabolic outcomes and controlled prospective human studies should be initiated and followed. We truly believe that experimental and clinical studies should include an assessment of intestinal barrier integrity, markers of the generalized inflammatory process (e.g., LPS), and the effect of gut microbiota modifications (prebiotics, probiotics, antibiotics) on metabolic side effects of SGAs. Lastly, studies in the field of metabolomics should also be the next step in such experiments to. Due to individual composition and function of gut microbiota, the content of the microbiome metabolites, e.g., SCFAs or secondary bile acids, which play an important role in metabolic and cardiovascular health, would comprehensively decipher the impact of SGA-induced dysbiosis on human metabolism (Alemán et al. 2018; Chambers et al. 2018).

## Electronic supplementary material

ESM 1 (DOCX 21 kb)
