# Supplementary material for: Second-generation antipsychotics and metabolism alterations: a systematic review of the role of the gut microbiome
Source: Psychopharmacology (Berl). 2018 Nov 20;236(5):1491–512. doi: 10.1007/s00213-018-5102-6 (PMC6598971; doi:10.1007/s00213-018-5102-6)
Supplement: Supplementary file 1 — (DOCX 21 kb) [file 213_2018_5102_MOESM1_ESM.docx]

Supporting Information:

Figure S1. Quality of rodent studies.

Figure S2. Risk of bias in rodent studies.
